# Oral hygiene management in critically ill patients: prevention of ventilator-associated pneumonia

**DOI:** 10.3389/fdmed.2026.1748329

**Published:** 2026-02-19

**Authors:** M. Martelli, A. Rosa, M. Miranda, R. Simone, M. Scarpati Cioffari di Castiglione, F. De Falco, M. Gargari, P. Bollero, F. Gianfreda

**Affiliations:** 1 Department of Clinical Sciences and Translational Medicine, University of Rome “Tor Vergata”, Rome, Italy; 2 Department of System Medicine, University of Rome “Tor Vergata”, Rome, Italy

**Keywords:** intensive Care Unit, mechanical ventilation, Oral—general health, Oral Hygiene, ventilator-associated pneumonia

## Abstract

**Background:**

Oral hygiene in critically ill patients in intensive care is essential for preventing infectious complications, particularly ventilator-associated pneumonia (VAP), which is one of the most severe and frequent complications in these units. The management of oral health can reduce bacterial load and the risk of respiratory infections.

**Materials and methods:**

This study analyzes oral hygiene management strategies in critically ill patients undergoing mechanical ventilation through a systematic literature review following the PRISMA-ScR method. Studies published from 2000 to 2023 were examined, focusing on the comparison between mechanical plaque removal interventions and the use of chlorhexidine against other measures.

**Results:**

The results highlight that the combined use of mechanical plaque removal and chlorhexidine significantly reduces the incidence of VAP and improves oral hygiene. The analysis of the evidence suggests that standardized protocols and a multidisciplinary approach, including collaboration with dental specialists, can optimize oral health in these critically ill patients.

**Discussion:**

The collected evidence underscores the importance of oral hygiene management not only to reduce the risk of VAP but also to improve the overall well-being of patients in intensive care. It is crucial to consider each patient's specific conditions, including comorbidities, to implement personalized interventions that minimize associated risks. Despite promising results, further investigation is needed regarding the optimization of oral hygiene techniques and the standardization of protocols to ensure uniformity in clinical practices.

**Conclusion:**

The findings support the importance of adequate oral hygiene management in intensive care to reduce the risk of respiratory infections, emphasizing the need for continued research and the development of evidence-based practices that enhance clinical outcomes.

## Highlights

Identify oral hygiene as a critical factor in preventing ventilator-associated pneumonia in intensive care patients.
Demonstrate the effectiveness of combining chlorhexidine and mechanical plaque removal in reducing infection rates.
Highlight the importance of standardized protocols and multidisciplinary collaboration in oral care management.
Suggest that dental interventions improve patient outcomes and reduce healthcare-associated complications.
Emphasize the clinical relevance of integrated oral hygiene strategies in critical care settings.

## Introduction

Intensive care is one of the most complex and delicate branches of medicine, dedicated to the treatment of critically ill patients with severe or potentially life-threatening conditions. These patients require constant monitoring and intensive treatments that include respiratory, hemodynamic, and pharmacological support. Severe clinical conditions and the prolonged use of medical devices, such as endotracheal tubes, profoundly alter the general and local physiological framework, including the oral cavity. This compromised environment favors the onset of various pathologies, particularly infectious ones, both locally (oral) and systemically (respiratory) (1).

Numerous studies highlight how patients admitted to intensive care units have a high incidence of oral cavity pathologies, often underestimated or inadequately treated due to the complexity of these individuals' general conditions. Among the most common issues is the alteration of salivary flow, which can lead to xerostomia, resulting in oral lesions and a greater predisposition to infections, such as those caused by Candida (2). Additionally, the decrease in immune defenses, due to pathological conditions and pharmacological treatments, makes patients more vulnerable to the development of caries and periodontal diseases. Furthermore, great attention should be paid to peri-implantitis, which can very easily lead to the formation of purulent inflammatory infiltrate (3).

Following a careful analysis of the most recent studies in the literature, it has emerged that critically ill patients have a high incidence of periodontal disease. Managing this aspect is crucial, as this condition can significantly affect overall health. Periodontitis could not only aggravate pre-existing conditions but also favor the development of new complications, such as lung infections, resulting from the migration of pathogens from the oral cavity to the respiratory tract (4).

Nosocomial pneumonias (HAP, Healthcare Associated Pneumoniae) account for about 25% of nosocomial infections in intensive care units (5). Nosocomial pneumonias are defined as lung infections that develop within 48 h of hospital admission and were not already incubating at the time of admission due to the composition of the oral microbiota, which undergoes a transformation towards more virulent organisms, increasing the risk of nosocomial infections (5).

Ventilator-associated pneumonia (VAP) develops primarily as a result of microaspiration of contaminated oropharyngeal secretions around the endotracheal tube cuff. Endotracheal intubation disrupts normal airway defenses, impairs cough reflexes, and facilitates the formation of biofilm on the surface of the tube, which serves as a reservoir for pathogenic microorganisms. Additional contributing factors include prolonged mechanical ventilation, supine positioning, sedation, and frequent airway manipulation (6).

Critically ill patients are particularly vulnerable to VAP due to the presence of multiple comorbidities such as chronic obstructive pulmonary disease, diabetes mellitus, cardiovascular disease, renal failure, malnutrition, and immunosuppression. These conditions, combined with altered immune responses and prolonged exposure to invasive devices and broad-spectrum antibiotics, significantly increase the risk of respiratory infections in the intensive care setting (6, 7).

Among the most common and feared nosocomial pneumonias (HAP, Hospital-Acquired Pneumonia) recorded in intensive care units, ventilator-associated pneumonia (VAP) is one of the most frequent and severe complications in intensive care units. VAP often develops due to bacterial colonization of the oral cavity and oropharynx, favored by poor oral hygiene and the use of ventilation devices that facilitate the migration of pathogens to the respiratory tract (5, 6).

Ventilator-associated respiratory infections represent a significant clinical challenge, with high rates of morbidity and mortality and are responsible for a considerable prolongation of hospital stays. This necessitates targeted preventive interventions, including optimal oral hygiene management. The decontamination of the oropharynx and the adoption of dental care protocols in intensive care are key elements to prevent the onset of respiratory complications. However, despite the growing attention to this issue, there remains ample room for improvement in the clinical practices adopted (5, 7, 8).

Numerous studies have demonstrated the effectiveness of preventive and therapeutic dental interventions in reducing respiratory infections, highlighting the need for greater integration of oral care within the management protocols of critically ill patients (5, 9, 10).

This article aims to systematically examine, through a scoping review, the possible dental management strategies for patients admitted to intensive care. The intent is to provide a comprehensive overview of current prevention and treatment practices, with particular attention to methodologies aimed at reducing the risk of respiratory infections, including VAP. The analysis of existing scientific literature will allow the identification of effective dental interventions, thereby contributing to improving the quality of care and clinical outcomes of patients admitted to intensive care units.

## Materials and methods

### Research strategy

The review was conducted according to the PRISMA-ScR methodology (Preferred Reporting Items for Systematic Reviews and Meta-Analyses extension for Scoping Reviews). The primary objective was to identify relevant studies on the management of oral hygiene in patients undergoing mechanical ventilation in intensive care, with particular attention to the prevention of ventilator-associated pneumonia (VAP). The quantitative section is organized around a main research question based on the PICOT framework, as explained below.

**Population**: Critically ill patients admitted to intensive care and undergoing mechanical ventilation.
**Intervention**: Plaque removal using toothbrushes, oral swabs, 0.12%–0.2% chlorhexidine, and povidone-iodine.
**Comparison:** Standard oral care or alternative oral hygiene protocols (e.g., chlorhexidine alone vs. placebo/standard care, toothbrushing/mechanical plaque removal alone, povidone-iodine, saline or sodium bicarbonate solutions), depending on the study design.
**Outcomes**: Improvement in oral hygiene assessed through the Brushing Observation Evaluation (BOE), the Clinical Pulmonary Infection Score (CPIS), and reduction in the incidence of ventilator-associated pneumonia (VAP).
**Study Type**: Randomized controlled trials (RCTs), observational studies, and case-control studies published between 2000 and 2023.

### Data sources and search strategies

The research was conducted using the following electronic databases: PubMed, Scopus, and Web of Science. The keywords used included: [oral health], [intensive care unit], [dental management], [ventilator-associated pneumonia], and [oral hygiene].

### Study selection

**Figure 1:**
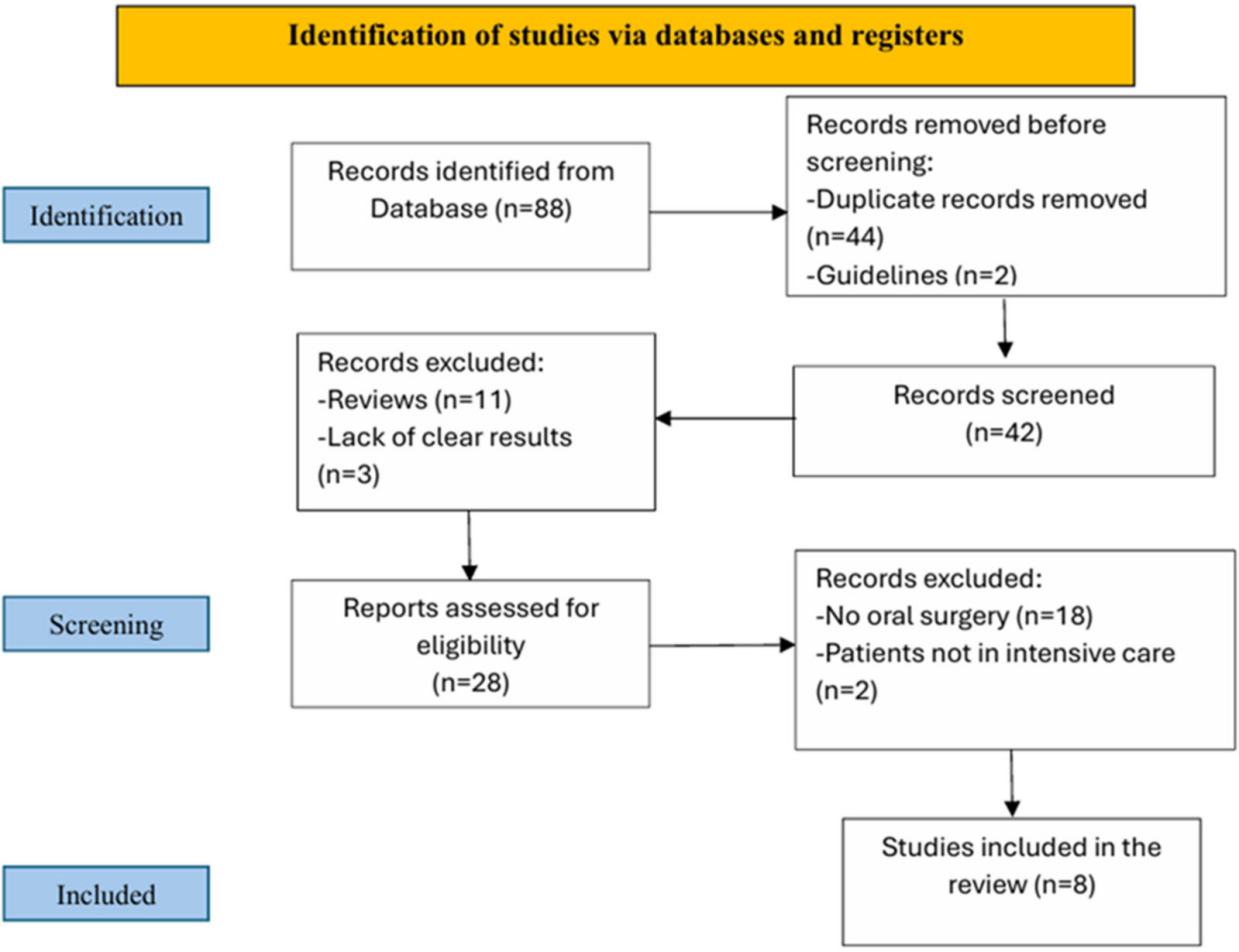
Selection process flow chart.

The study selection was performed by two independent reviewers. Each reviewer examined the titles and abstracts of potential articles and applied the inclusion criteria. Disagreements were resolved through discussion or by involving a third reviewer. Duplicate articles were eliminated during the selection process (Figure 1).

### Data extraction and analysis

The extracted data included: author, year of publication, study type, population characteristics, type of intervention, and main results regarding the prevention of VAP (Table 1).

**Table 1:** Main characteristics of the studies.

Studies included	Study Design	Year of publication	Population	Sex M/W	Age
Bellissimo-Rodrigues	randomised clinical trial	2018	Control group (*n* = 127)	66/61 (control)	60.1 ± 17.5 (control);
			Intervention group (*n* = 127)	67/60 (intervention)	53.4 ± 18.3 (intervention)
Bergmans	prospective, randomized, double-blind, placebo-controlled study	2001	Control group (A = 78; B = 61)	53/25 (control A)	58,1 ± 16,4 (control A)
			Intervention group (*n* = 87)	47/14 (control B)	58,7 ± 16,7 (control B)
				59/28 (intervention)	56,6 ± 19 (intervention)
Choi	Randomized Controlled Trial	2022	Control group (*n* = 28)	19/9 (control)	57.43 ± 16.94(control)
			Intervention group (*n* = 29)	20/9 (intervention)	60.62 ± 15.67 (intervention)
de Lacerda Vidal	randomized study	2017	Control group (*n* = 108)	54/54 (control)	63,2 ± 14,5 (control) 59,4 ± 14,5 (ntervention)
			Intervention group (*n* = 105)	51/54 (intervention)	
Am	randomised clinical trial	2016	Control group (*n* = 50)	Not specified	51,5 ± 1,3 (control)
			Intervention group (*n* = 50)		52,1 ± 1,2 (intervention)
Kes	Randomized controlled study	2021	Control group (*n* = 28)	16/12 (control)	77,37 ± 10,1 (control)
			Intervention group (*n* = 29)	18/11 (intervention)	72,79 ± 12 (Intervention)
Tsuda	Randomized controlled study	2020	Control group (*n* = 7)	4/3 (control)	65,8 (control)
			Intervention group (*n* = 16)	10/6 (intervention)	63,5 (Intervention)
Tuon	Prospective, randomised, controlled study	2017	Control group (*n* = 8)	4/4 (control)	48,2 (control)
			Intervention group (*n* = 8)	5/3 (intervention)	53,1 (Intervention)

## Results

The literature search generated 88 references: of these, 44 duplicate articles and 2 guidelines were removed before screening. 42 articles were screened, from which 11 reviews and 3 articles without clear results were excluded. 28 articles were assessed for eligibility, of which 18 were excluded because they did not include oral cavity treatments and 2 because they did not include intensive care patients. In the end, 8 studies were included in the review (10–17).

The studied population included patients admitted to intensive care, with a portion of them undergoing mechanical ventilation. The average age of the patients ranged from 48 to 77 years, with a predominance of male patients (approximately 57%).

The evaluated oral hygiene interventions included plaque removal using toothbrushes, oral swabs, 0.12%–0.2% chlorhexidine, and povidone-iodine. Mechanical plaque removal was performed in 5 studies (10–13, 17), while the use of chlorhexidine was evaluated in 7 studies (10–15, 17). The use of povidone-iodine was less common, present in only one study (16). Interventions, outcomes, and complications from the selected articles have been reported in a table (Table 2).

**Table 2:** Intervention, outcame and complication reported.

Studies included	Year of publication	Intervention	Outcame	Complication
Bellissimo-Rodriguez	2018	Brushing teeth with 0.12% chlorhexidine gel (control); dental treatment provided by a dentist (intervention)	Respiratory tract infections (RTIs) prevented approximately 56% of lower respiratory tract infections in the intervention group compared to the control group.	Mucosal irritation 3–15;
				Intra-oral bleeding 5–4;
				All adverse events 8–17.
Bergmans	2001	Topical antimicrobial prophylaxis (gentamicin/colistin/vancomycin 2% in Oracase, every 6 h)	Incidences of VAP were 10% in study patients, 31% in Group A, and 23% in Group B patients	Recurrent VAP
				Study patients: 2
				Group A: 2
				Group B: 1
Choi	2022	The intervention group received professional oral hygiene care and rinses with 0.12% CHX, while the control group received general oral hygiene care.	No cases of VAP occurred in the intervention group. The levels of Staphylococcus aureus and Klebsiella pneumoniae decreased significantly.	In the control group, there were two cases of VAP. The study had a relatively high dropout rate (about 34%), due to various factors such as deaths, extubations, and transfers.
De Lacerda Vidal	2017	0.12% chlorhexidine oral solution (control group); tooth brushing with 0.12% chlorhexidine gel (intervention group)	Development of VAP	Deaths
			Control group: 28	Control group: 27
			Intervention group: 17	Intervention group: 20
Haghighi	2016	Control group: Brush teeth once a day and rinse with a chlorhexidine solution twice a day.	Patients in the intervention group showed a significant improvement in oral health compared to the control group. The mucosal-plaque index also improved significantly in the intervention group. The incidence of ventilator-associated pneumonia was reduced in the intervention group. In the intervention group, the average BOAS score decreased from 10.7 on the first day to 8.9 on the fifth day, while in the control group, it increased from 10.46 on the first day to 11.3.	Deaths
		Intervention group: Adjust endotracheal tube pressure; brush teeth and gums with a children's toothbrush; rinse with saline solution; spray chlorhexidine on teeth, tongue, and gums, followed by deep suction. Hydrate lips and mouth with vitamin A + D.		Intervention Group: 2 patients died.
				Control Group: 3 patients died.
				The incidence of VAP was reduced in the intervention group compared to the control group, although the difference was not statistically significant.
Kes	2021	The intervention group received oral hygiene three times a day using a 0.12% chlorhexidine gluconate solution. The control group received oral hygiene three times a day using sodium bicarbonate.	In the CHX group, the incidence of VAP was significantly lower. There was a lower frequency of oropharyngeal colonization in the CHX group. Improvements in BOAG were observed in the CHX group.	In the CHX group, 2 patients developed VAP. In the placebo group, 5 patients developed VAP.
Tsuda	2020	Daily oral care with 3% hydrogen peroxide and irrigation with 200 mL of tap water. In the intervention group, 10% povidone-iodine (5 mL) was applied topically.	The application of povidone-iodine significantly reduced bacterial growth in the intervention group for up to 3 hours after application and did not disturb the balance of the oral microbiota or promote the growth of resistant microorganisms.	The study acknowledges some limitations, including the small sample size and the limited follow-up duration (only up to 3 hours), which may have restricted the observation of any long-term complications.
Tuon	2017	The intervention group received an oral rinse with 15 mL of a 2% chlorhexidine digluconate solution. The solution was brushed onto the gums, oral mucosa, and tongue twice a day. Patients in the placebo group received an oral rinse with a 0.9% NaCl solution.	The CHX group had a lower incidence of methicillin-resistant Staphylococcus aureus (MRSA) compared whitthe placebo	Four patients in the CHX group and two patients in the placebo group developed VAP.

### Effectiveness of interventions

Mechanical Plaque Removal: Mechanical plaque removal showed a significant reduction in the incidence of ventilator-associated pneumonia (VAP) in 3 studies (10–12). However, two studies did not report a significant reduction, indicating variability in the results. One study found that the use of a toothbrush reduced the incidence of VAP and the duration of mechanical ventilation (13).
Use of Chlorhexidine: The only use of 0.12%–0.2% chlorhexidine was associated with a significant reduction in the incidence of VAP in 1 study (17). Chlorhexidine proved effective in reducing the total number of bacteria present in the mouth, including Staphylococcus aureus and Klebsiella pneumoniae (12). In a further study, Bambi S. et al. reiterate that in intubated patients who do not receive adequate oral care, there is a proliferation of gram-negative bacteria such as MRSA, Pseudomonas aeruginosa, Staphylococcus aureus, Streptococcus pneumoniae, Acinetobacter baumanii, Hemophilus influenzae, considered potential etiological agents of VAP (7). According to Kishimoto and Urade, the physical elimination of microorganisms can improve the effectiveness of chlorhexidine against residual bacteria or their reproduction (18–21).
Use of Povidone-Iodine: The use of povidone-iodine showed a reduction in bacterial growth in the oral cavity, but there is no conclusive evidence of its effectiveness in reducing the incidence of VAP (16).

Following these interventions, it was observed how the reduction in the incidence of VAP was the primary outcome evaluated. Studies that combined mechanical plaque removal with the use of chlorhexidine showed a more significant reduction in the incidence of VAP compared to the use of chlorhexidine alone (17). In addition, it is evident how the use of these methods has improved the oral hygiene of the patients. This was also evaluated using the Brushing Observation Evaluation (BOE) (12) and the Clinical Pulmonary Infection Score (CPIS) in 3 studies (12, 14, 15), while the Beck Oral Assessment Scale (BOAS) was used in only one study (14). Combined interventions of mechanical removal and the use of chlorhexidine showed the best results in terms of oral hygiene (11).

Despite the methods used to improve oral conditions in intensive care patients with mechanical ventilation, the major complication that can be encountered, despite the interventions, is the onset of VAP. Some studies reported patient deaths; however, it is essential to emphasize that these were due to concomitant pathologies and the severity of critical illness, rather than (being related to oral health or) oral hygiene measures (13, 14).

## Discussion

The findings of our scoping review emphasize the critical importance of oral hygiene management in intensive care patients, particularly for preventing ventilator-associated pneumonia (VAP). VAP remains one of the most severe and frequent complications in intensive care units, contributing to high morbidity and mortality rates. Oral hygiene interventions, such as the use of chlorhexidine and mechanical plaque removal, have shown a significant impact in reducing VAP incidence. This aligns with previous research highlighting chlorhexidine's effectiveness in lowering oral bacterial load and preventing respiratory infections (5, 6).

Mechanical Plaque Removal: Combining mechanical plaque removal with chlorhexidine use yielded the most favorable outcomes in reducing VAP incidence and improving oral hygiene. While three studies (10–12) demonstrated a significant decrease in VAP incidence due to mechanical plaque removal, two studies did not find a significant reduction, indicating variability in results. This suggests the need for further research to optimize these protocols and better understand the conditions under which these interventions are most effective.
Use of Chlorhexidine: Chlorhexidine has shown promising results in reducing VAP incidence and bacterial colonization in the respiratory tract, making it a key component of oral hygiene protocols for intensive care patients (10–15, 17).
Use of Povidone-Iodine: The lack of evidence in the use of povidone-iodine suggests the necessity for additional studies to evaluate povidone-iodine's potential as an alternative or complement to chlorhexidine (16).
Improvement in Oral Hygiene: Combined interventions of mechanical removal and chlorhexidine use showed the best results in maintaining oral hygiene. This suggests that an integrated approach, combining various oral hygiene techniques, may be more effective in maintaining a healthy oral environment and preventing infections (22–27).

Despite the methods used to enhance oral conditions in mechanically ventilated intensive care patients, VAP remains a significant complication (28, 29). Despite the methods used to enhance oral conditions in mechanically ventilated intensive care patients, VAP remains a significant complication (28, 29). Some studies reported patient deaths; however, it is crucial to note that these were due to concomitant pathologies and the severity of critical illness rather than (being related to oral health or) oral hygiene measures (13, 14). Additionally, several limitations should be considered:

Sample Size: Some studies had relatively small sample sizes, which may have affected the statistical significance of the results.
Follow-up Duration: The follow-up duration was limited in some studies, potentially restricting the observation of long-term complications.
Protocol Variability: The variability in oral hygiene protocols used makes direct comparison of results challenging. This suggests the need to standardize protocols to improve comparability and reproducibility of results.
Implementation and integration: Few studies provided detailed information on how oral care knowledge and dental interventions were integrated into ICU policies, procedures, staff training, and interprofessional communication pathways. This may influence adherence and effectiveness and should be considered when interpreting outcomes and designing future protocols.

Based on the evidence gathered, we recommend integrating standardized oral hygiene protocols in intensive care units. The use of chlorhexidine, both as a mouthwash and gel, should be combined with mechanical plaque removal to maximize effectiveness (17). In addition, implementation should be embedded in a multidisciplinary ICU framework (e.g., nurses, intensivists, infection prevention teams, respiratory therapists, and dental professionals), supported by clear policies, procedures, and communication tools to ensure consistent delivery of oral care.

To address the temporal scope of the evidence base, we conducted an updated search to identify studies published after 2023. Recent syntheses provide additional context and highlight ongoing debate regarding the optimal antiseptic strategy. A 2024 network meta-analysis of randomized trials suggested that toothbrushing, particularly when combined with 0.12% chlorhexidine, ranked highly for VAP prevention, although the certainty of evidence was low (30). Conversely, another 2024 network meta-analysis reported no significant benefit of chlorhexidine at any concentration for preventing VAP and did not support its routine use (31). Moreover, implementation-oriented studies published in 2024–2025 emphasize that standardized, intensive oral care and interprofessional training may be key drivers of improved outcomes, irrespective of the specific antiseptic used (32–34).

Several studies have investigated the role of oral hygiene interventions in preventing ventilator-associated pneumonia in critically ill patients. Previous randomized controlled trials have demonstrated that mechanical plaque removal combined with chlorhexidine use significantly reduces oral bacterial load and the incidence of VAP compared with standard oral care alone. These findings are consistent with those reported by Bergmans et al. and de Lacerda Vidal et al., who showed a reduction in VAP incidence when structured oral hygiene protocols were implemented in mechanically ventilated patients.

More recent studies have emphasized the importance of professional dental involvement in intensive care units, highlighting improved oral health outcomes and a reduction in respiratory infections when dental care is integrated into multidisciplinary ICU teams. Our findings align with this evidence, supporting the concept that oral hygiene should be considered an essential component of VAP prevention bundles rather than an optional supportive measure.

The high prevalence of comorbidities among critically ill patients further supports the need for targeted preventive strategies. Poor oral health, periodontal disease, and increased oral bacterial load may act as modifiable risk factors for VAP, particularly in patients with compromised immune responses. Therefore, structured oral hygiene protocols should be considered an integral component of VAP prevention bundles in intensive care units.

It is essential to continue promoting research in this field to identify the best oral hygiene practices and develop evidence-based protocols. Future studies should focus on larger samples and long-term follow-up to evaluate the effectiveness and safety of oral hygiene interventions in intensive care patients. Additionally, exploring the use of new technologies and materials could further enhance oral hygiene and infection prevention.

This scoping review has several limitations that should be acknowledged. First, the number of available studies focusing specifically on oral hygiene interventions in mechanically ventilated intensive care patients remains limited. Second, the included studies show substantial heterogeneity in terms of study design, sample size, oral hygiene protocols, frequency of interventions, and outcome measures, which limits direct comparability. Additionally, follow-up periods were often short, potentially underestimating long-term outcomes and complications. Finally, some studies included small patient cohorts, which may have reduced statistical power. These limitations highlight the need for further large-scale, well-designed randomized controlled trials with standardized protocols.

Overall, the present scoping review provides an updated synthesis of the available evidence on oral hygiene management in critically ill patients, reinforcing the clinical relevance of structured oral care protocols and supporting their integration into routine intensive care practice.

## Conclusion

The management of oral hygiene in critically ill patients admitted to intensive care is a fundamental aspect for preventing infectious complications, particularly ventilator-associated pneumonia (VAP). The literature review highlighted that the use of chlorhexidine, both as a mouthwash and gel, is effective in reducing the incidence of VAP due to its bactericidal and bacteriostatic properties. However, the effectiveness of chlorhexidine is maximized when combined with mechanical plaque removal, suggesting that an integrated approach is essential to achieve the best results.

The use of non-absorbable antibiotics has shown potential in reducing the oral microbial load but has not demonstrated a significant impact on patient mortality, thus limiting its use as a standard of care. Additionally, the adoption of oral hygiene protocols that include oropharyngeal decontamination and the use of appropriate tools, such as small-headed toothbrushes with soft bristles, is crucial for improving oral hygiene and reducing the risk of respiratory infections.

The collected evidence underscores the importance of a multidisciplinary approach that includes the involvement of specialized dentists within intensive care teams. Their expertise is essential for performing necessary procedures such as caries treatment, scaling, root planing, and drainage of oral abscesses. Interprofessional collaboration is crucial for ensuring optimal and holistic care, improving patient well-being, and reducing healthcare costs associated with prolonged hospital stays and infectious complications.

In conclusion, the integration of effective oral hygiene practices and the adoption of a multidisciplinary approach can significantly improve clinical outcomes for critically ill patients, reducing the morbidity and mortality associated with nosocomial infections. It is essential to continue promoting research and the implementation of evidence-based oral care protocols to optimize the management of patients in intensive care.

## Data Availability

The original contributions presented in the study are included in the article/Supplementary Material, further inquiries can be directed to the corresponding authors.
